# Human Langerhans Cells with Pro-inflammatory Features Relocate within Psoriasis Lesions

**DOI:** 10.3389/fimmu.2018.00300

**Published:** 2018-02-22

**Authors:** Liv Eidsmo, Elisa Martini

**Affiliations:** ^1^Department of Medicine Solna, Karolinska Institutet, Solna, Sweden; ^2^Department of Dermatology, Karolinska University Hospital, Stockholm, Sweden

**Keywords:** Langerhans cells, human, psoriasis, microenvironment, inflammation, Langerhans cell function, Langerhans cell localization

## Abstract

Psoriasis is a common skin disease that presents with well-demarcated patches of inflammation. Recurrent disease in fixed areas of the skin indicates a localized disease memory that is preserved in resolved lesions. In line with such concept, the involvement of tissue-resident immune cells in psoriasis pathology is increasingly appreciated. Langerhans cells (LCs) are perfectly placed to steer resident T cells and local tissue responses in psoriasis. Here, we present an overview of the current knowledge of LCs in human psoriasis, including findings that highlight pro-inflammatory features of LCs in psoriasis lesions. We also review the literature on conflicting data regarding LC localization and functionality in psoriasis. Our review highlights that further studies are needed to elucidate the molecular mechanisms that drive LCs functionality in inflammatory diseases.

## Self-Renewing LCs form a Cellular Network in Healthy Epidermis

The human skin forms a sophisticated barrier in which resident immune cells orchestrate immune responses against foreign antigens, while maintaining tolerance to commensals (1). In focal inflammatory skin diseases, tissue homeostasis is unevenly disturbed, and patches of intense inflammation are surrounded by apparently normal skin. Local alterations of resident immune cells are increasingly appreciated in these diseases. Langerhans cells (LCs) form a stable pool of professional antigen-presenting cells resident in healthy epidermis with distinct ontogeny and phenotypes compared to dermal dendritic cells (DCs) (2, 3). The CFS-1 receptor ligand IL-34, abundantly produced by keratinocytes, is crucial for LC development within the skin (4), whereas LC residency is strongly dependent on the constitutive expression of TGF-β (5). In contrast, replenishment of dermal subsets of DCs is dependent on the differentiation of circulating precursors and is driven by the tyrosine kinase FLT3 ligand (6, 7). LCs predominately self-renew within murine (8) and human skin, with donor-derived LCs detected up to 10 years after human hand transplantation (9, 10). However, in murine models of inflammation and infection, short-lived and bone marrow-derived CCR2-expressing myeloid precursors fill up the epidermal niche following LC depletion, indicating heterogeneity within the pool of LCs in resolved skin lesions (8, 11). Human LCs form a network capable of sensing the entire skin surface (12) and comprise 2–4% of epidermal cells with a surface density of 500–1,000 cells per mm^2^ (13, 14). Apart from their ability to sense danger and present antigens, the function of human LCs remains debated after more than a century of studies in healthy and diseased conditions.

Functional studies in murine models have provided fundamental insights into LC biology in different settings of tissue immunity and inflammation. However, profound anatomical and immunological differences are obvious when comparing murine and human skin. Human epidermis comprises several layers of keratinocytes (14) and is dominated by interfollicular epithelium, whereas murine epidermis comprises 2–3 cell layers and is covered by dense hair follicles (1). Although many aspects of LC biology and functionality are comparable between mouse and man (15), epidermal lymphocyte populations differ, with αβ T cells populating human epidermis and dendritic epidermal T cells and γδ T cells dominating the murine epidermis (16). Finally, albeit inflammatory models have provided valuable information on LC biology, the full complexity of human inflammatory skin diseases cannot be captured in murine models (17).

## Psoriasis Occurs in Fixed Patches of the Skin

Psoriasis is one example of a focal inflammatory skin disease where disturbance of LC biology has been reported. Psoriasis affects 2–3% of the human population and typically presents with macroscopic well-demarcated, red, and scaly plaques. Genetic predisposition increases the risk of psoriasis (18), and several psoriasis-associated genes are linked to the immune system. In particular, *HLA-Cw6* is strongly associated with psoriasis and genome-wide association studies link psoriasis to polymorphisms of genes belonging to MHC class I pathway (*ERAP1*), IL-23 signaling pathway (*IL12B, IL23A*, and *IL23R*), cytokines pathways and Th17 polarization (*STAT3*), or NF-kB pathway (*CARD14*) (19). Epidermal hyperplasia, focal immune cell infiltration, and vascular changes dominate the microscopic disturbances in affected sites, whereas non-lesional and resolved skin at large appears normal. Contemporary immunological findings support the idea that psoriasis plaques are maintained by interactions between aberrantly differentiated keratinocytes and immune cells, both resident and recruited. Myeloid and lymphoid immune cells including T cells, innate lymphoid cells, inflammatory DCs, and neutrophils accumulate in psoriasis lesions and produce disease-driving effector molecules such as IL-23, TNF, IL-17, IL-22, granzyme A, and IFN-γ *in situ* (20–34). Both genetic and therapeutic studies imply that cytokines originating from DCs are involved in psoriasis pathogenesis. The influx of several subsets of inflammatory DCs into psoriasis lesions is discussed in several recent reviews (35, 36). In contrast, few studies have characterized LCs in psoriasis. Nevertheless, these few studies have shed some light on the complexity and plasticity of human LCs. As of yet, less can be concluded regarding pathologic consequences of such LC alterations.

## Microenvironmental Alterations Associated with Psoriasiform Inflammation Impact on LC Functionality

LCs sense the external environment and the microbiota covering the human body through dendrites protruding all the way to the apical part of epidermis (12). Compared to dermal DCs, LCs express fewer Toll-like receptors (TLRs) (37–39), which indicates impaired capacity to respond to TLR signaling (39). It is plausible that LCs maintain tolerance to commensals during homeostatic conditions (40). Within psoriasis lesions, LCs are exposed to a complex plethora of inflammatory signals that might affect the expression pattern and the activation threshold of TLRs. In contrast to atopic dermatitis, the few available reports on the psoriasis microbiome have not been able to highlight striking alterations from healthy skin (41–45). Higher resolution analysis using shot-gun metagenomics, ideally combined with genetic and transcriptomic analysis, may shed light on psoriasis–dysbiosis. It would be of particular interest to investigate the fungal microbiome in psoriasis, taken that IL-17 is associated with fungal responses (46). Another source of external influence on LCs functionality is systemic medication. Angiotensin II inhibitors, a common treatment for hypertension, dampen TGF-β signaling and reduce the density of LCs in human skin (5). In a number of case reports, losartan is implicated as a triggering factor for psoriasis (47, 48), and it would be interesting to investigate the activation status and functionality of LCs in such patients.

**Figure 1:**
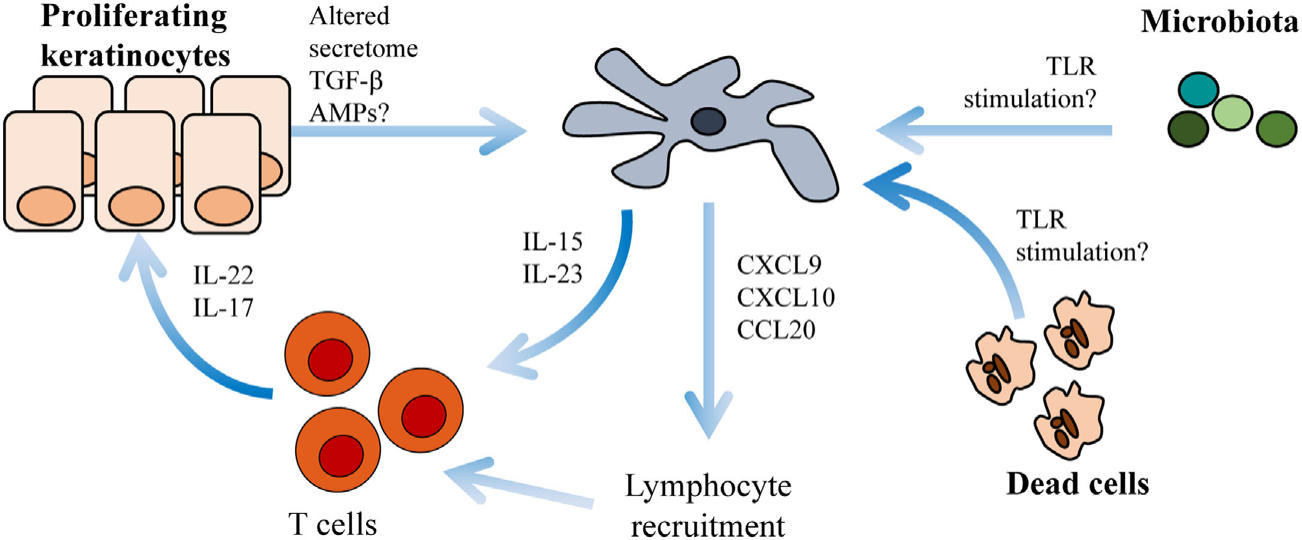
Langerhans cells (LCs) cross-talk with keratinocytes and T cells within psoriasis plaques. Environmental triggers such as altered microbiota, necrotic cells or antimicrobial peptides (AMPs) activate LCs to produce IL-15, IL-23 (31, 56), CXCL9, CXCL10, and CCL20 (57). IL-15 and IL-23 induce T cell activation of IL-22 and IL-17. CXCL9, CXCL10, and CCL20 are chemotactic molecules important for further lymphocyte recruitment.

Activated keratinocytes represent another LC-trigger in the skin milieu (49, 50). Both keratinocytes and T cells secrete the psoriasis triggering cytokine granulocyte–macrophage colony-stimulating factor (GM-CSF) (51). GM-CSF induces LC maturation and exacerbates their stimulatory capacity (52). It is plausible that activated keratinocytes interact with LCs in evolving psoriasis lesions. In psoriasis plaques, keratinocytes upregulate the antimicrobial peptide LL-37 (53) that theoretically should activate LCs (54). Activated LCs could potentially present antigens *in situ* to T cells infiltrating the skin. IL-22 and IL-17 produced by T cells in psoriasis plaques amplify the production of the antimicrobial peptide LL-37 in keratinocytes (55), thereby perpetuating this potential inflammatory loop (Figure 1).

## Altered Localization of LCs within Psoriasis Lesion

**Figure 2:**
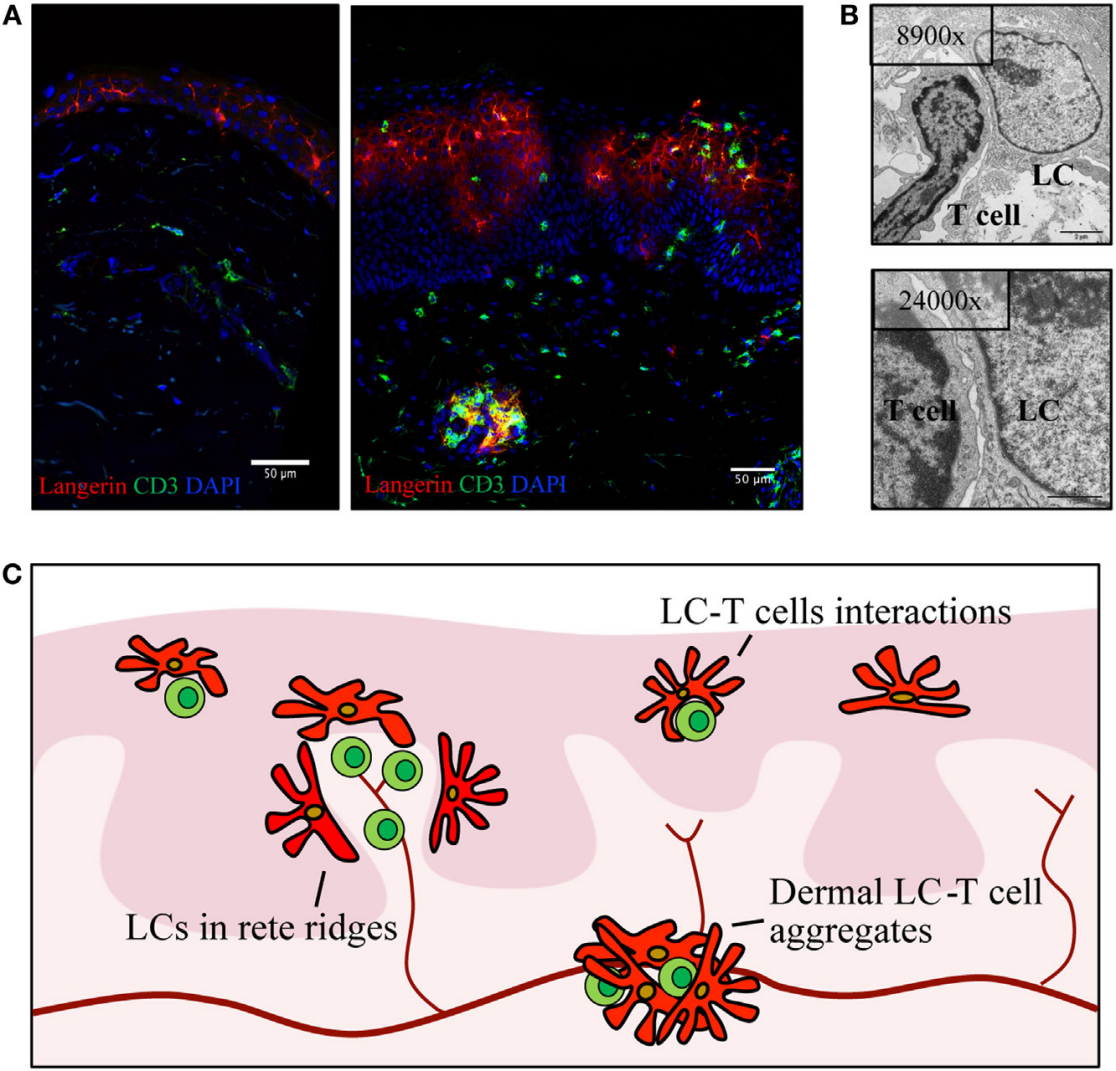
Langerhans cells (LCs) relocate within psoriatic lesions and translocate into dermis. **(A)** Confocal image of healthy skin (left) and active psoriasis (right). LCs are visualized by Langerin (red) and T cells by CD3 (green) with mixed cellular aggregates present in both epidermis and dermis of psoriasis skin. **(B)** Transmission electron microscopy images depicting an example of LCs–T cell interaction in psoriasis epidermis. **(C)** Schematic highlighting localization of LCs (red) in active psoriasis skin, in relation to T cells (green) and blood vessels (dark red). Illustrations from the Eidsmo laboratory of previously published data (31, 75).

Conflicting data regarding the density of LCs in psoriasis have been debated since the seventies with reports detecting increased (57, 59, 60), decreased (61–63), or stable (22, 31, 64–66) densities of LCs in psoriasis-afflicted epidermis. Interindividual variation in LC density is considerable in healthy and psoriasis-affected subjects, and thus, the variable results may be a consequence of underpowered studies. In addition, local redistribution of LCs and shared surface markers with inflammatory DCs complicate the assessment of LC density within psoriasis plaques. In active psoriasis, LCs co-localize with T cells and inflammatory DCs in epidermal aggregates and relocate within epidermis to the basement membrane and to the apical part of the dedifferentiated epidermis (Figures 2A–C) (67). To add a further layer of complexity, increased density of LCs in perilesional skin, close to the border of active psoriasis lesions, has been reported (60, 68, 69), and the conflicting results obtained by different investigators might be affected by the location within the psoriatic lesion that was sampled. Increased (60, 64, 68) or similar (70) numbers of LCs in non-involved psoriasis skin in comparison to healthy skin have been described. In resolved psoriasis lesions, the LC number is reduced after PUVA treatment (64, 71, 72) and increased during anti-TNF treatment (69, 73, 74). Despite the experimental challenges in enumerating LCs, the wealth of conflicting studies suggests dynamics in the survival or migrational patterns of LCs in psoriasis.

More than 10 years ago, elegant studies from the Griffith laboratory identified migrational impairment in LCs in response to IL-1β and TNF injection in non-lesional psoriasis skin (70). Reduction in the number of epidermal LCs was measured 2–4 h after injection, and follow-up studies further stratified LC responses to the time of onset of psoriasis (58, 70, 76). Reduced migration of LCs toward inflammatory cytokines is attributed to the IL-17-induced keratinocyte secretome (77). A possible consequence of this migrational impairment is the accumulation of LCs in the dermis (67, 78–82), where they form dermal aggregates with T cells outside lymphatic vessels (Figures 2A–C). The localization of LCs in dermis and basal epidermis, adjacent to the basement membrane in areas where dermal immune cells are entering the epidermis, raises questions on the nature of interactions between LCs and other inflammatory cells, questions that at large remain to be answered.

## Functional Profiling Reveals Inflammatory Properties of LCs in Psoriasis Plaques

Functional studies on human LCs in psoriasis lesions are scarce in comparison to the wealth of studies that have investigated the properties of blood-derived lesional immune cells. In line with the pro-inflammatory microenvironment within the psoriasis lesions (83), and despite the tolerogenic potential of LCs in healthy skin (40), LCs seem to play an active role in sustaining the inflammation in psoriasis. Transcriptional profiling of LCs sorted from lesional psoriasis revealed expression of several immune cell attracting chemokines including *CXCL1* and *CXCL10* and inflammatory chemokines such as *CCL18* and *CCL20* (57). In contrast, LCs derived from atopic dermatitis preferentially expressed *CCL17* and *CCL20*, underlying the disease specificity of LC function. In a recent publication, a subset of myeloid cells expressing CD5 promoting induction of IL-22, IFNγ, TNF, and granzyme B in mixed lymphocyte reaction assays was enriched in psoriasis epidermis (84). We and others have directly shown that that LCs from psoriatic lesions produce IL-23 following TLR activation (31, 56), thereby directly linking LCs to the pathogenic IL-23/IL-17 axis. Conversely, lesional LCs also display increased mRNA levels for several tolerogenic factors, including IDO-1, PD-L1, and PD-L2 (31), which complicates interpretations of their role in chronic psoriasis plaque. New techniques are needed to fully understand the overall role of LCs in psoriasis, but current data point toward an active participation in shaping the local inflammatory milieu (Figure 1).

## Local Antigen Presentation in Psoriasis Lesions

Systemic administration of T cell-depleting antibodies temporarily normalizes psoriasis pathology in human patients (85). Epidermal T cells accumulate (86, 87) and co-localize with both LCs and inflammatory DCs within psoriasis lesions (Figure 2B) (31). The impressive inflammatory profile of epidermal T cells (30, 33, 34, 88) could result from *in situ* stimulation by epidermal LCs and DCs. Indeed, several studies have shown that DCs derived from psoriasis lesions sustain the inflammation by producing TNF, iNOS, and IL-23 (20, 22–24, 26, 29, 35, 75). Furthermore, lesional DCs are capable of activating allogenic T cells and induce production of IL-17, IL-22, and IFN-γ (29, 57, 80, 89). Intriguingly, LCs from psoriasis skin show similar ability to stimulate allogenic T cells compared to LCs sorted from atopic dermatitis-affected skin (57). Several autoantigens are proposed to be important to maintain psoriasis (53, 90–92), but the polyclonal pool of pro-inflammatory T cells in psoriasis plaques complicates the concept of psoriasis as a purely autoimmune disease (92, 93). Lipid antigens are presented by CD1a, highly expressed on LCs and on inflammatory DCs. The presence of CD1a-restricted T cells polarized to IL-17 and IL-22 production in lesional psoriasis (91) is indicative of *in situ* antigen presentation of LCs to T cells, but formal proof of such events remains to be shown in human settings.

## LCs in Resolved Skin Show Potential to Maintain Pathogenic Resident T Cells

Therapies for psoriasis range from topical treatments to UV therapy and systemic immunomodulatory treatments. Biologics targeting cell-to-cell signaling through TNF or the IL-23/IL-17 pathway have revolutionized the clinical management of severe psoriasis. This range of different treatment strategies offers a possibility to investigate LC biology in different settings of resolved psoriasis (94, 95). UV light induces LC migration from the epidermis, and a reduction in the number of LCs in the skin has been noted after UV treatment (59, 72). TNF inhibitors alter the balance of resident and infiltrating DCs in both epidermis and dermis (69, 73, 74). Despite complete resolution of macroscopic disease, the local transcriptome remains dysregulated following both UVB and anti-TNF treatment (96, 97), and resident T cells poised to produce IL-17A and IL-22 accumulate in resolved lesions (30, 88). LCs sorted from resolved lesions after successful treatment with UVB therapy retain elevated *IL15* expression, whereas LCs from anti-TNF-treated lesions display residual *IL23* expression. Furthermore, LCs sorted from resolved lesions during TNF treatment, unlike healthy LCs, are able to respond to TLR stimulation with IL-23 production (31). These findings, together with their placement in close contact with T cells within active lesions (Figure 2B), put LCs both in the right place and perfectly equipped to induce IL-17 and IL-22 production in IL-23R-positive epidermal resident T cells (88).

## Lessons Learnt from LC Biology in Murine Models of Psoriasis

Psoriasis is restricted to the human species; nevertheless, several murine models have been developed to mimic psoriasiform inflammation (17). These models provide an attractive tool to further explore findings from human studies in an *in vivo* setting (Table 1). Several mouse models support the idea that LCs have a pathogenic role in acute disease. In early studies using the flaky skin mouse, where mice develop scaling and vessel abnormalities as a consequence of an autosomal recessive mutation of the *Ttc7* gene, the number of LCs increases in acute disease (98) and is reduced after administration of an IL-1β-neutralizing antibody (99). In the IMQ model, the density of epidermal LCs is reduced and coupled with enhanced LC emigration to the skin-draining lymph nodes (100). More strikingly, data from IMQ-induced psoriasiform inflammation show that LCs produce pro-inflammatory cytokines necessary to activate pathogenic T cells (56, 101, 102), whereas other studies focus on the role of dermal DCs in driving psoriasiform inflammation (103–106). Conversely, recent work attribute LCs a protective role with elevated levels of IL-10 mRNA and upregulation of PD-L1 in the *Jun^f/f^JunB^f/f^K5cre^ER^* (DKO*) mouse (62). Moreover, after long-term application of IMQ, LCs are important to control the influx of neutrophils into the epidermis (81), indicating that LCs relocated to the border between epidermis and dermis may act as gate keepers that influence on epidermal tissue homeostasis. Collectively, it appears that the lack of clarity on LCs in human psoriasis is mirrored in the mouse models.

**Table 1:** Alteration of LCs in human psoriasis and in mouse models.

LCs	Observed effect		Observation in humans	Observation in murine models	Mouse model
Phenotype	Epidermal density of LCs	Increase	Baker et al. (59), Komine et al. (60), Fujita et al. (57)	Sundberg et al. (98), Schön et al. (99), Singh et al. (105), Xiao et al. (101)	Flaky skin mouse, IL-23 injection, IMQ

		Decrease	Lisi (63), Bos et al. (61), Glitzner et al. (62)	Suzuki et al. (100), Glitzner et al. (62)	IMQ

		Stable	Gommans et al. (65), Czernielewski et al. (64), Gunther et al. (66), Martini et al. (31)	–	–

	IL-23 and inflammatory chemokines production		Fujita et al. (57), Sweeney et al. (56), Martini et al. (31)	Yoshiki et al. (102), Sweeney et al. (56), Xiao et al. (101)	IMQ

	IL-10 and PD-L1 expression		–	Glitzner et al. (62)	DKO*

Function	Migratory capacity	Increased	–	Suzuki et al. (100), Glitzner et al. (62), Xiao et al. (101)	IMQ, DKO*

		Impaired	Cumberbatch et al. (70), Shaw et al. (76)	–	–

	Enhanced T cell stimulatory ability		Fujita et al. (57)	Yoshiki et al. (102), Xiao et al. (101)	IMQ

**dDCs**	**Observed effect**		**Observation in humans**	**Observation in murine models**	**Mouse model**

Phenotype	Density of dDCs	Increase	Summarized by Haniffa et al. (6), Jariwala (35), Kim et al. (36)	Glitzner et al. (62), van der Fits (107), Terhorst et al. (81), Singh et al. (105)	DKO*, IMQ, IL-23 injection

	Pro-inflammatory cytokine profile (production of IL-23, TNF, iNOS)		Wohn et al. (106), Massot et al. (103), Singh et al. (105)	IMQ, IL-23 injection

Function	Enhanced T cell stimulatory ability		Wohn et al. (106), Massot et al. (103)	IMQ

*LC, Langerhans cell*.

## Challenges in Phenotypic and Functional Studies of LC in Psoriasis Pathogenesis

Characterization of LCs in psoriasis lesions was initially performed by immunohistochemistry using markers such as HLA-DR, CD1a, and s100 proteins (59, 61, 108, 109). However, inflammatory epidermal DCs share many of the cellular markers previously used to define LCs (22, 31, 57). In humans, in both healthy skin and inflamed skin, the most reliable markers for epidermal LCs are Birbeck granules and Langerin. Langerin has an extracellular domain and an intracellular domain located within the Birbeck granules (110), therefore choosing the right antibody is essential to optimize cell sorting. Although low expression of CD1a is detected on inflammatory DCs, flow cytometry can separate CD1a-bright LCs and CD1a-dim inflammatory DCs. Despite reliable protocols to sort LCs *ex vivo* (111), functional studies require substantial numbers of viable cells which complicates the analysis of clinical material. Decreased LC viability following tissue isolation procedures or even short-term culture (112–114) further impedes the study of human LC functionality. Instead, LC-like cells differentiated from blood CD34^+^ precursors have been used. Although these cells share some properties with primary LCs, they display a mature phenotype (115) with profound differential transcriptomic profiles (116). It is important to bear these methodologic challenges in mind when assessing the wealth of sometimes conflicting reports on LC biology.

## Conclusion and Future Perspectives

In psoriasis lesions, LCs relocate both within the epidermis and to the dermis. Their localization in close contact with lesional T cells indicates that LCs may participate in focal immunopathology. Indeed, LCs are poised to produce IL-23 in active and resolved psoriasis lesions. However, despite considerable efforts in a multitude of models and settings, the role of LCs in psoriasis pathogenesis remains to be shown. With the current speed of development of experimental techniques combined with the wealth of novel immunotherapies, ample opportunities to fully elucidate the involvement of LCs in psoriasiform inflammation should present themselves over the years to come.

## Author Contributions

LE and EM planned the outline, reviewed the literature, and wrote this paper together. EM prepared figures and tables.

## Conflict of Interest Statement

The authors declare that the research was conducted in the absence of any commercial or financial relationships that could be construed as a potential conflict of interest.

## References

[1] Pasparakis M Haase I Nestle FO. Mechanisms regulating skin immunity and inflammation. Nat Rev Immunol (2014) 14:289–301. 10.1038/nri3646 24722477

[2] Ginhoux F Merad M. Ontogeny and homeostasis of Langerhans cells. Immunol Cell Biol (2010) 88:387–92. 10.1038/icb.2010.38 20309014

[3] Merad M Sathe P Helft J Miller J Mortha A. The dendritic cell lineage: ontogeny and function of dendritic cells and their subsets in the steady state and the inflamed setting. Annu Rev Immunol (2013) 31:563–604. 10.1146/annurev-immunol-020711-074950 23516985 PMC3853342

[4] Wang Y Colonna M. Interkeukin-34, a cytokine crucial for the differentiation and maintenance of tissue resident macrophages and Langerhans cells. Eur J Immunol (2014) 44:1575–81. 10.1002/eji.201344365 24737461 PMC4137395

[5] Mohammed J Beura LK Bobr A Astry B Chicoine B Kashem SW Stromal cells control the epithelial residence of DCs and memory T cells by regulated activation of TGF-beta. Nat Immunol (2016) 17:414–21. 10.1038/ni.3396 26901152 PMC5135085

[6] Haniffa M Gunawan M Jardine L. Human skin dendritic cells in health and disease. J Dermatol Sci (2015) 77:85–92. 10.1016/j.jdermsci.2014.08.012 25301671 PMC4728191

[7] Breton G Lee J Zhou YJ Schreiber JJ Keler T Puhr S Circulating precursors of human CD1c+ and CD141+ dendritic cells. J Exp Med (2015) 212:401–13. 10.1084/jem.20141441 25687281 PMC4354370

[8] Merad M Manz MG Karsunky H Wagers A Peters W Charo I Langerhans cells renew in the skin throughout life under steady-state conditions. Nat Immunol (2002) 3:1135–41. 10.1038/ni852 12415265 PMC4727838

[9] Kanitakis J Morelon E Petruzzo P Badet L Dubernard JM. Self-renewal capacity of human epidermal Langerhans cells: observations made on a composite tissue allograft. Exp Dermatol (2011) 20:145–6. 10.1111/j.1600-0625.2010.01146.x 20707812

[10] Kanitakis J Petruzzo P Dubernard JM Turnover of epidermal Langerhans’ cells. N Engl J Med (2004) 351:2661–2. 10.1056/NEJM200412163512523 15602033

[11] Eidsmo L Allan R Caminschi I van Rooijen N Heath WR Carbone FR. Differential migration of epidermal and dermal dendritic cells during skin infection. J Immunol (2009) 182:3165–72. 10.4049/jimmunol.0802950 19234214

[12] Kubo A Nagao K Yokouchi M Sasaki H Amagai M. External antigen uptake by Langerhans cells with reorganization of epidermal tight junction barriers. J Exp Med (2009) 206:2937–46. 10.1084/jem.20091527 19995951 PMC2806471

[13] Bauer J Bahmer FA Worl J Neuhuber W Schuler G Fartasch M. A strikingly constant ratio exists between Langerhans cells and other epidermal cells in human skin. A stereologic study using the optical disector method and the confocal laser scanning microscope. J Invest Dermatol (2001) 116:313–8. 10.1046/j.1523-1747.2001.01247.x 11180009

[14] Kanitakis J. Anatomy, histology and immunohistochemistry of normal human skin. Eur J Dermatol (2002) 12:390–9; quiz 400–1. 12095893

[15] Griffiths CE Dearman RJ Cumberbatch M Kimber I. Cytokines and Langerhans cell mobilisation in mouse and man. Cytokine (2005) 32:67–70. 10.1016/j.cyto.2005.07.011 16153855

[16] Mestas J Hughes CC. Of mice and not men: differences between mouse and human immunology. J Immunol (2004) 172:2731–8. 10.4049/jimmunol.172.5.2731 14978070

[17] Gudjonsson JE Johnston A Dyson M Valdimarsson H Elder JT. Mouse models of psoriasis. J Invest Dermatol (2007) 127:1292–308. 10.1038/sj.jid.5700807 17429444

[18] Bowcock AM The genetics of psoriasis and autoimmunity. Annu Rev Genomics Hum Genet (2005) 6:93–122. 10.1146/annurev.genom.6.080604.162324 16124855

[19] Harden JL Krueger JG Bowcock AM. The immunogenetics of Psoriasis: a comprehensive review. J Autoimmun (2015) 64:66–73. 10.1016/j.jaut.2015.07.008 26215033 PMC4628849

[20] Brunner PM Koszik F Reininger B Kalb ML Bauer W Stingl G. Infliximab induces downregulation of the IL-12/IL-23 axis in 6-sulfo-LacNac (slan)+ dendritic cells and macrophages. J Allergy Clin Immunol (2013) 132:1184–93.e8. 10.1016/j.jaci.2013.05.036 23890755

[21] Cai Y Shen X Ding C Qi C Li K Li X Pivotal role of dermal IL-17-producing gammadelta T cells in skin inflammation. Immunity (2011) 35:596–610. 10.1016/j.immuni.2011.08.001 21982596 PMC3205267

[22] Guttman-Yassky E Lowes MA Fuentes-Duculan J Whynot J Novitskaya I Cardinale I Major differences in inflammatory dendritic cells and their products distinguish atopic dermatitis from psoriasis. J Allergy Clin Immunol (2007) 119:1210–7. 10.1016/j.jaci.2007.03.006 17472813

[23] Hansel A Gunther C Ingwersen J Starke J Schmitz M Bachmann M Human slan (6-sulfo LacNAc) dendritic cells are inflammatory dermal dendritic cells in psoriasis and drive strong TH17/TH1 T-cell responses. J Allergy Clin Immunol (2011) 127:787–94.e1–e9. 10.1016/j.jaci.2010.12.009 21377044

[24] Lee E Trepicchio WL Oestreicher JL Pittman D Wang F Chamian F Increased expression of interleukin 23 p19 and p40 in lesional skin of patients with psoriasis vulgaris. J Exp Med (2004) 199:125–30. 10.1084/jem.20030451 14707118 PMC1887731

[25] Lin AM Rubin CJ Khandpur R Wang JY Riblett M Yalavarthi S Mast cells and neutrophils release IL-17 through extracellular trap formation in psoriasis. J Immunol (2011) 187:490–500. 10.4049/jimmunol.1100123 21606249 PMC3119764

[26] Teunissen MB Zheng L de Groot M de Rie MA Fine JS Chen SC. Rise in dermal CD11c+ dendritic cells associates with early-stage development of psoriatic lesions. Arch Dermatol Res (2012) 304:443–9. 10.1007/s00403-012-1231-7 22438166 PMC3401310

[27] Teunissen MBM Munneke JM Bernink JH Spuls PI Res PCM Te Velde A Composition of innate lymphoid cell subsets in the human skin: enrichment of NCR(+) ILC3 in lesional skin and blood of psoriasis patients. J Invest Dermatol (2014) 134:2351–60. 10.1038/jid.2014.146 24658504

[28] Yawalkar N Tscharner GG Hunger RE Hassan AS. Increased expression of IL-12p70 and IL-23 by multiple dendritic cell and macrophage subsets in plaque psoriasis. J Dermatol Sci (2009) 54:99–105. 10.1016/j.jdermsci.2009.01.003 19264456

[29] Zaba LC Fuentes-Duculan J Eungdamrong NJ Abello MV Novitskaya I Pierson KC Psoriasis is characterized by accumulation of immunostimulatory and Th1/Th17 cell-polarizing myeloid dendritic cells. J Invest Dermatol (2009) 129:79–88. 10.1038/jid.2008.194 18633443 PMC2701224

[30] Hijnen D Knol EF Gent YY Giovannone B Beijn SJ Kupper TS CD8(+) T cells in the lesional skin of atopic dermatitis and psoriasis patients are an important source of IFN-gamma, IL-13, IL-17, and IL-22. J Invest Dermatol (2013) 133:973–9. 10.1038/jid.2012.456 23223131 PMC3835628

[31] Martini E Wiken M Cheuk S Gallais Serezal I Baharom F Stahle M Dynamic changes in resident and infiltrating epidermal dendritic cells in active and resolved psoriasis. J Invest Dermatol (2017) 137:865–73. 10.1016/j.jid.2016.11.033 28011143

[32] Tonel G Conrad C Laggner U Di Meglio P Grys K McClanahan TK Cutting edge: a critical functional role for IL-23 in psoriasis. J Immunol (2010) 185:5688–91. 10.4049/jimmunol.1001538 20956338 PMC3776381

[33] Cheuk S Martini E Bergh K Chang D Rethi B Stahle M Granzyme A potentiates chemokine production in IL-17-stimulated keratinocytes. Exp Dermatol (2017) 26:824–7. 10.1111/exd.13284 28094457

[34] Cheuk S Schlums H Gallais Serezal I Martini E Chiang SC Marquardt N CD49a expression defines tissue-resident CD8+ T cells poised for cytotoxic function in human skin. Immunity (2017) 46:287–300. 10.1016/j.immuni.2017.01.009 28214226 PMC5337619

[35] Jariwala SP. The role of dendritic cells in the immunopathogenesis of psoriasis. Arch Dermatol Res (2007) 299:359–66. 10.1007/s00403-007-0775-4 17680257 PMC1978540

[36] Kim TG Kim DS Kim HP Lee MG. The pathophysiological role of dendritic cell subsets in psoriasis. BMB Rep (2014) 47:60–8. 10.5483/BMBRep.2014.47.2.014 24411465 PMC4163895

[37] Renn CN Sanchez DJ Ochoa MT Legaspi AJ Oh CK Liu PT TLR activation of Langerhans cell-like dendritic cells triggers an antiviral immune response. J Immunol (2006) 177:298–305. 10.4049/jimmunol.177.1.298 16785525

[38] Takeuchi J Watari E Shinya E Norose Y Matsumoto M Seya T Down-regulation of toll-like receptor expression in monocyte-derived Langerhans cell-like cells: implications of low-responsiveness to bacterial components in the epidermal Langerhans cells. Biochem Biophys Res Commun (2003) 306:674–9. 10.1016/S0006-291X(03)01022-2 12810071

[39] van der Aar AM Sylva-Steenland RM Bos JD Kapsenberg ML de Jong EC Teunissen MB. Loss of TLR2, TLR4, and TLR5 on Langerhans cells abolishes bacterial recognition. J Immunol (2007) 178:1986–90. 10.4049/jimmunol.178.4.1986 17277101

[40] Seneschal J Clark RA Gehad A Baecher-Allan CM Kupper TS. Human epidermal Langerhans cells maintain immune homeostasis in skin by activating skin resident regulatory T cells. Immunity (2012) 36:873–84. 10.1016/j.immuni.2012.03.018 22560445 PMC3716276

[41] Fahlen A Engstrand L Baker BS Powles A Fry L. Comparison of bacterial microbiota in skin biopsies from normal and psoriatic skin. Arch Dermatol Res (2012) 304:15–22. 10.1007/s00403-011-1189-x 22065152

[42] Gao Z Tseng CH Strober BE Pei Z Blaser MJ. Substantial alterations of the cutaneous bacterial biota in psoriatic lesions. PLoS One (2008) 3:e2719. 10.1371/journal.pone.0002719 18648509 PMC2447873

[43] Grice EA Kong HH Conlan S Deming CB Davis J Young AC Topographical and temporal diversity of the human skin microbiome. Science (2009) 324:1190–2. 10.1126/science.1171700 19478181 PMC2805064

[44] Paulino LC Tseng CH Strober BE Blaser MJ. Molecular analysis of fungal microbiota in samples from healthy human skin and psoriatic lesions. J Clin Microbiol (2006) 44:2933–41. 10.1128/JCM.00785-06 16891514 PMC1594634

[45] Tett A Pasolli E Farina S Truong DT Asnicar F Zolfo M Unexplored diversity and strain-level structure of the skin microbiome associated with psoriasis. NPJ Biofilms Microbiomes (2017) 3:14. 10.1038/s41522-017-0022-5 28649415 PMC5481418

[46] Acosta-Rodriguez EV Rivino L Geginat J Jarrossay D Gattorno M Lanzavecchia A Surface phenotype and antigenic specificity of human interleukin 17-producing T helper memory cells. Nat Immunol (2007) 8:639–46. 10.1038/ni1467 17486092

[47] Lamba G Palaniswamy C Singh T Shah D Lal S Vinnakota R Psoriasis induced by losartan therapy: a case report and review of the literature. Am J Ther (2011) 18:e78–80. 10.1097/MJT.0b013e3181c6c0c2 20027103

[48] Marquart-Elbaz C Grosshans E Lipsker D Lipsker D Sartans, angiotensin II receptor antagonists, can induce psoriasis. Br J Dermatol (2002) 147:617–8. 10.1046/j.1365-2133.2002.48848.x 12207619

[49] Gudjonsson JE Ding J Johnston A Tejasvi T Guzman AM Nair RP Assessment of the psoriatic transcriptome in a large sample: additional regulated genes and comparisons with in vitro models. J Invest Dermatol (2010) 130:1829–40. 10.1038/jid.2010.36 20220767 PMC3128718

[50] Mee JB Johnson CM Morar N Burslem F Groves RW. The psoriatic transcriptome closely resembles that induced by interleukin-1 in cultured keratinocytes: dominance of innate immune responses in psoriasis. Am J Pathol (2007) 171:32–42. 10.2353/ajpath.2007.061067 17591951 PMC1941577

[51] Braun-Falco O Dermatology. Berlin, NY: Springer (2000).

[52] Jonuleit H Knop J Enk AH. Cytokines and their effects on maturation, differentiation and migration of dendritic cells. Arch Dermatol Res (1996) 289:1–8. 10.1007/s004030050144 9017128

[53] Lande R Botti E Jandus C Dojcinovic D Fanelli G Conrad C The antimicrobial peptide LL37 is a T-cell autoantigen in psoriasis. Nat Commun (2014) 5:5621. 10.1038/ncomms6621 25470744

[54] Ogawa Y Kawamura T Matsuzawa T Aoki R Gee P Yamashita A Antimicrobial peptide LL-37 produced by HSV-2-infected keratinocytes enhances HIV infection of Langerhans cells. Cell Host Microbe (2013) 13:77–86. 10.1016/j.chom.2012.12.002 23332157

[55] Sakabe J Umayahara T Hiroike M Shimauchi T Ito T Tokura Y. Calcipotriol increases hCAP18 mRNA expression but inhibits extracellular LL37 peptide production in IL-17/IL-22-stimulated normal human epidermal keratinocytes. Acta Derm Venereol (2014) 94:512–6. 10.2340/00015555-1775 24419155

[56] Sweeney CM Russell SE Malara A Kelly G Hughes R Tobin AM Human ss-defensin 3 and its mouse ortholog murine ss-defensin 14 activate langerhans cells and exacerbate psoriasis-like skin inflammation in mice. J Invest Dermatol (2016) 136:723–7. 10.1016/j.jid.2015.12.011 27015459

[57] Fujita H Shemer A Suarez-Farinas M Johnson-Huang LM Tintle S Cardinale I Lesional dendritic cells in patients with chronic atopic dermatitis and psoriasis exhibit parallel ability to activate T-cell subsets. J Allergy Clin Immunol (2011) 128:574–82.e1–e12. 10.1016/j.jaci.2011.05.016 21704361

[58] Eaton LH Chularojanamontri L Ali FR Theodorakopoulou E Dearman RJ Kimber I Guttate psoriasis is associated with an intermediate phenotype of impaired Langerhans cell migration. Br J Dermatol (2014) 171:409–11. 10.1111/bjd.12960 24628096

[59] Baker BS Swain AF Griffiths CE Leonard JN Fry L Valdimarsson H. Epidermal T lymphocytes and dendritic cells in chronic plaque psoriasis: the effects of PUVA treatment. Clin Exp Immunol (1985) 61:526–34. 3878241 PMC1577286

[60] Komine M Karakawa M Takekoshi T Sakurai N Minatani Y Mitsui H Early inflammatory changes in the “perilesional skin” of psoriatic plaques: is there interaction between dendritic cells and keratinocytes? J Invest Dermatol (2007) 127:1915–22. 10.1038/sj.jid.5700799 17446902

[61] Bos JD Hulsebosch HJ Krieg SR Bakker PM Cormane RH. Immunocompetent cells in psoriasis. In situ immunophenotyping by monoclonal antibodies. Arch Dermatol Res (1983) 275:181–9. 10.1007/BF00510050 6604503

[62] Glitzner E Korosec A Brunner PM Drobits B Amberg N Schonthaler HB Specific roles for dendritic cell subsets during initiation and progression of psoriasis. EMBO Mol Med (2014) 6:1312–27. 10.15252/emmm.201404114 25216727 PMC4287934

[63] Lisi P Investigation on Langerhans cells in pathological human epidermis. Acta Derm Venereol (1973) 53:425–8. 4129586

[64] Czernielewski J Juhlin L Shroot S Brun P. Langerhans’ cells in patients with psoriasis: effect of treatment with PUVA, PUVA bath, etretinate and anthralin. Acta Derm Venereol (1985) 65:97–101. 2408430

[65] Gommans JM van Hezik SJ van Huystee BE. Flow cytometric quantification of T6-positive cells in psoriatic epidermis after PUVA and methotrexate therapy. Br J Dermatol (1987) 116:661–6. 10.1111/j.1365-2133.1987.tb05899.x 3496110

[66] Gunther C Starke J Zimmermann N Schakel K. Human 6-sulfo LacNAc (slan) dendritic cells are a major population of dermal dendritic cells in steady state and inflammation. Clin Exp Dermatol (2012) 37:169–76. 10.1111/j.1365-2230.2011.04213.x 22188261

[67] Clarke LE Helm KF Hennessy J Bruggeman RD Clarke JT. Dermal dendritic cells in psoriasis, nummular dermatitis, and normal-appearing skin. J Am Acad Dermatol (2012) 66:98–105. 10.1016/j.jaad.2010.12.001 21669473

[68] Alshenawy HA Hasby EA. Immunophenotyping of dendritic cells in lesional, perilesional and distant skin of chronic plaque psoriasis. Cell Immunol (2011) 269:115–9. 10.1016/j.cellimm.2011.03.015 21470599

[69] Gordon KB Bonish BK Patel T Leonardi CL Nickoloff BJ. The tumour necrosis factor-alpha inhibitor adalimumab rapidly reverses the decrease in epidermal Langerhans cell density in psoriatic plaques. Br J Dermatol (2005) 153:945–53. 10.1111/j.1365-2133.2005.06816.x 16225604

[70] Cumberbatch M Singh M Dearman RJ Young HS Kimber I Griffiths CE. Impaired Langerhans cell migration in psoriasis. J Exp Med (2006) 203:953–60. 10.1084/jem.20052367 16567387 PMC2118293

[71] Erkin G Ugur Y Gurer CK Asan E Korkusuz P Sahin S Effect of PUVA, narrow-band UVB and cyclosporin on inflammatory cells of the psoriatic plaque. J Cutan Pathol (2007) 34:213–9. 10.1111/j.1600-0560.2006.00591.x 17302604

[72] Ree K. Reduction of Langerhans cells in human epidermis during PUVA therapy: a morphometric study. J Invest Dermatol (1982) 78:488–92. 10.1111/1523-1747.ep12510257 7086168

[73] Hendriks AG van der Velden HM Wolberink EA Seyger MM Schalkwijk J Zeeuwen PL The effect of adalimumab on key drivers in the pathogenesis of psoriasis. Br J Dermatol (2014) 170:571–80. 10.1111/bjd.12705 24640989

[74] Piaserico S Zattra E Michelotto A Alaibac M. Effects of TNF-alpha inhibitors on the number of epidermal Langerhans cells in uninvolved skin of psoriatic patients: a pilot study. Acta Histochem (2013) 115:767–9. 10.1016/j.acthis.2013.02.013 23566554

[75] Lowes MA Chamian F Abello MV Fuentes-Duculan J Lin SL Nussbaum R Increase in TNF-alpha and inducible nitric oxide synthase-expressing dendritic cells in psoriasis and reduction with efalizumab (anti-CD11a). Proc Natl Acad Sci U S A (2005) 102:19057–62. 10.1073/pnas.0509736102 16380428 PMC1323218

[76] Shaw FL Cumberbatch M Kleyn CE Begum R Dearman RJ Kimber I Langerhans cell mobilization distinguishes between early-onset and late-onset psoriasis. J Invest Dermatol (2010) 130:1940–2. 10.1038/jid.2010.57 20237494

[77] Eaton LH Mellody KT Pilkington SM Dearman RJ Kimber I Griffiths CEM Impaired Langerhans’ cell migration in psoriasis is due to an altered keratinocyte phenotype induced by interleukin-17. Br J Dermatol (2017). 10.1111/bjd.16172 29194565

[78] Ramirez-Bosca A Martinez-Ojeda L Valcuende-Cavero F Castells-Rodellas A. A study of local immunity in psoriasis. Br J Dermatol (1988) 119:587–95. 10.1111/j.1365-2133.1988.tb03469.x 2905164

[79] Reynolds NJ Yi JY Fisher GJ Cooper KD Voorhees JJ Griffiths CE. Down-regulation of Langerhans cell protein kinase C-beta isoenzyme expression in inflammatory and hyperplastic dermatoses. Br J Dermatol (1995) 133:157–67. 10.1111/j.1365-2133.1995.tb02611.x 7547380

[80] Fujita H Nograles KE Kikuchi T Gonzalez J Carucci JA Krueger JG. Human Langerhans cells induce distinct IL-22-producing CD4+ T cells lacking IL-17 production. Proc Natl Acad Sci U S A (2009) 106:21795–800. 10.1073/pnas.0911472106 19996179 PMC2799849

[81] Terhorst D Chelbi R Wohn C Malosse C Tamoutounour S Jorquera A Dynamics and transcriptomics of skin dendritic cells and macrophages in an imiquimod-induced, biphasic mouse model of psoriasis. J Immunol (2015) 195:4953–61. 10.4049/jimmunol.1500551 26466959

[82] Raaby L Rosada C Langkilde A Lauridsen KL Vinter H Ommen P Langerhans cell markers CD1a and CD207 are the most rapidly responding genes in lesional psoriatic skin following adalimumab treatment. Exp Dermatol (2017) 26:804–10. 10.1111/exd.13304 28109175

[83] Baliwag J Barnes DH Johnston A. Cytokines in psoriasis. Cytokine (2015) 73:342–50. 10.1016/j.cyto.2014.12.014 25585875 PMC4437803

[84] Korenfeld D Gorvel L Munk A Man J Schaffer A Tung T A type of human skin dendritic cell marked by CD5 is associated with the development of inflammatory skin disease. JCI Insight (2017) 2:96101. 10.1172/jci.insight.96101 28931765 PMC5621884

[85] Prinz J Braun-Falco O Meurer M Daddona P Reiter C Rieber P Chimaeric CD4 monoclonal antibody in treatment of generalised pustular psoriasis. Lancet (1991) 338:320–1. 10.1016/0140-6736(91)90464-Z 1677143

[86] Conrad C Boyman O Tonel G Tun-Kyi A Laggner U de Fougerolles A Alpha1beta1 integrin is crucial for accumulation of epidermal T cells and the development of psoriasis. Nat Med (2007) 13:836–42. 10.1038/nm1605 17603494

[87] Res PC Piskin G de Boer OJ van der Loos CM Teeling P Bos JD Overrepresentation of IL-17A and IL-22 producing CD8 T cells in lesional skin suggests their involvement in the pathogenesis of psoriasis. PLoS One (2010) 5:e14108. 10.1371/journal.pone.0014108 21124836 PMC2991333

[88] Cheuk S Wiken M Blomqvist L Nylen S Talme T Stahle M Epidermal Th22 and Tc17 cells form a localized disease memory in clinically healed psoriasis. J Immunol (2014) 192:3111–20. 10.4049/jimmunol.1302313 24610014 PMC3962894

[89] Lowes MA Kikuchi T Fuentes-Duculan J Cardinale I Zaba LC Haider AS Psoriasis vulgaris lesions contain discrete populations of Th1 and Th17 T cells. J Invest Dermatol (2008) 128:1207–11. 10.1038/sj.jid.5701213 18200064

[90] Arakawa A Siewert K Stohr J Besgen P Kim SM Ruhl G Melanocyte antigen triggers autoimmunity in human psoriasis. J Exp Med (2015) 212:2203–12. 10.1084/jem.20151093 26621454 PMC4689169

[91] Cheung KL Jarrett R Subramaniam S Salimi M Gutowska-Owsiak D Chen YL Psoriatic T cells recognize neolipid antigens generated by mast cell phospholipase delivered by exosomes and presented by CD1a. J Exp Med (2016) 213:2399–412. 10.1084/jem.20160258 27670592 PMC5068234

[92] Matos TR O’Malley JT Lowry EL Hamm D Kirsch IR Robins HS Clinically resolved psoriatic lesions contain psoriasis-specific IL-17-producing alphabeta T cell clones. J Clin Invest (2017) 4031–41. 10.1172/JCI93396 28945199 PMC5663366

[93] Harden JL Hamm D Gulati N Lowes MA Krueger JG. Deep sequencing of the T-cell receptor repertoire demonstrates polyclonal T-cell infiltrates in psoriasis. F1000Res (2015) 4:460. 10.12688/f1000research.6756.1 26594339 PMC4648215

[94] Boehncke WH Schon MP. Psoriasis. Lancet (2015) 386:983–94. 10.1016/S0140-6736(14)61909-7 26025581

[95] Hueber W Patel DD Dryja T Wright AM Koroleva I Bruin G Effects of AIN457, a fully human antibody to interleukin-17A, on psoriasis, rheumatoid arthritis, and uveitis. Sci Transl Med (2010) 2:52ra72. 10.1126/scitranslmed.3001107 20926833

[96] Goldminz AM Suarez-Farinas M Wang AC Dumont N Krueger JG Gottlieb AB. CCL20 and IL22 messenger RNA expression after adalimumab vs methotrexate treatment of psoriasis: a randomized clinical trial. JAMA Dermatol (2015) 151:837–46. 10.1001/jamadermatol.2015.0452 25946554 PMC5788701

[97] Suarez-Farinas M Fuentes-Duculan J Lowes MA Krueger JG. Resolved psoriasis lesions retain expression of a subset of disease-related genes. J Invest Dermatol (2011) 131:391–400. 10.1038/jid.2010.280 20861854 PMC3021088

[98] Sundberg JP Boggess D Sundberg BA Beamer WG Shultz LD. Epidermal dendritic cell populations in the flaky skin mutant mouse. Immunol Invest (1993) 22:389–401. 10.3109/08820139309063417 8406628

[99] Schön M Behmenburg C Denzer D Schon MP. Pathogenic function of IL-1 beta in psoriasiform skin lesions of flaky skin (fsn/fsn) mice. Clin Exp Immunol (2001) 123:505–10. 10.1046/j.1365-2249.2001.01421.x 11298140 PMC1906010

[100] Suzuki H Wang B Shivji GM Toto P Amerio P Tomai MA Imiquimod, a topical immune response modifier, induces migration of Langerhans cells. J Invest Dermatol (2000) 114:135–41. 10.1046/j.1523-1747.2000.00833.x 10620129

[101] Xiao C Zhu Z Sun S Gao J Fu M Liu Y Activation of Langerhans cells promotes the inflammation in imiquimod-induced psoriasis-like dermatitis. J Dermatol Sci (2017) 85:170–7. 10.1016/j.jdermsci.2016.12.003 27964879

[102] Yoshiki R Kabashima K Honda T Nakamizo S Sawada Y Sugita K IL-23 from Langerhans cells is required for the development of imiquimod-induced psoriasis-like dermatitis by induction of IL-17A-producing gammadelta T cells. J Invest Dermatol (2014) 134:1912–21. 10.1038/jid.2014.98 24569709

[103] Massot B Michel ML Diem S Ohnmacht C Latour S Dy M TLR-induced cytokines promote effective proinflammatory natural Th17 cell responses. J Immunol (2014) 192:5635–42. 10.4049/jimmunol.1302089 24808372

[104] Singh K Gatzka M Peters T Borkner L Hainzl A Wang H Reduced CD18 levels drive regulatory T cell conversion into Th17 cells in the CD18hypo PL/J mouse model of psoriasis. J Immunol (2013) 190:2544–53. 10.4049/jimmunol.1202399 23418628

[105] Singh TP Zhang HH Borek I Wolf P Hedrick MN Singh SP Monocyte-derived inflammatory Langerhans cells and dermal dendritic cells mediate psoriasis-like inflammation. Nat Commun (2016) 7:13581. 10.1038/ncomms13581 27982014 PMC5171657

[106] Wohn C Ober-Blobaum JL Haak S Pantelyushin S Cheong C Zahner SP Langerin(neg) conventional dendritic cells produce IL-23 to drive psoriatic plaque formation in mice. Proc Natl Acad Sci U S A (2013) 110:10723–8. 10.1073/pnas.1307569110 23754427 PMC3696803

[107] van der Fits L Mourits S Voerman JS Kant M Boon L Laman JD Imiquimod-induced psoriasis-like skin inflammation in mice is mediated via the IL-23/IL-17 axis. J Immunol (2009) 182(9):5836–45. 10.4049/jimmunol.0802999 19380832

[108] Heng MC Kloss SG. Cell interactions in psoriasis. Arch Dermatol (1985) 121:881–7. 10.1001/archderm.1985.01660070071018 2409930

[109] Bieber T Ring J Braun-Falco O. Comparison of different methods for enumeration of Langerhans cells in vertical cryosections of human skin. Br J Dermatol (1988) 118:385–92. 10.1111/j.1365-2133.1988.tb02432.x 3355780

[110] Valladeau J Ravel O Dezutter-Dambuyant C Moore K Kleijmeer M Liu Y Langerin, a novel C-type lectin specific to Langerhans cells, is an endocytic receptor that induces the formation of Birbeck granules. Immunity (2000) 12:71–81. 10.1016/S1074-7613(00)80160-0 10661407

[111] Gunawan M Jardine L Haniffa M. Isolation of human skin dendritic cell subsets. Methods Mol Biol (2016) 1423:119–28. 10.1007/978-1-4939-3606-9_8 27142012

[112] Bjercke S Elgo J Braathen L Thorsby E. Enriched epidermal Langerhans cells are potent antigen-presenting cells for T cells. J Invest Dermatol (1984) 83:286–9. 10.1111/1523-1747.ep12340417 6090538

[113] Simon JC Dittmar HC de Roche R Wilting J Christ B Schopf E. Rapid purification of human Langerhans cells using paramagnetic microbeads. Exp Dermatol (1995) 4:155–61. 10.1111/j.1600-0625.1995.tb00239.x 7551563

[114] Vremec D Hansen J Strasser A Acha-Orbea H Zhan Y O’Keeffe M Maintaining dendritic cell viability in culture. Mol Immunol (2015) 63:264–7. 10.1016/j.molimm.2014.07.011 25081090

[115] Klechevsky E Morita R Liu M Cao Y Coquery S Thompson-Snipes L Functional specializations of human epidermal Langerhans cells and CD14+ dermal dendritic cells. Immunity (2008) 29:497–510. 10.1016/j.immuni.2008.07.013 18789730 PMC2688399

[116] Harman AN Bye CR Nasr N Sandgren KJ Kim M Mercier SK Identification of lineage relationships and novel markers of blood and skin human dendritic cells. J Immunol (2013) 190:66–79. 10.4049/jimmunol.1200779 23183897

